# Differential expression profile of mRNAs, lncRNAs, and circRNAs reveals potential molecular mechanism in breast cancer

**DOI:** 10.1042/BSR20220645

**Published:** 2022-07-29

**Authors:** Yuan Li, Chiseng Lei, Yude Xie, Jie Zhang, Ningxia Wang, Weili He, Shaohua Qu

**Affiliations:** 1 Department of Breast Surgery, The First Affiliated Hospital of Jinan University, Jinan University, Guangzhou, China; 2 Department of Reproductive Medical Center, Guangdong Women and Children Hospital, Guangzhou, China

**Keywords:** breast cancer, ceRNA, fatty acid metabolism, molecular mechanism, RNA sequencing

## Abstract

In recent years, breast cancer attracts more and more attention because of its high incidence. To explore the molecular functions and mechanisms, we performed RNA sequencing on the tumor tissues and their paired normal tissues from three breast cancer patients. By differential expression analysis, we found 3764 differentially expressed (DE) mRNAs, 5416 DE lncRNAs, and 148 DE circRNAs. Enrichment analysis suggested that the DE lncRNAs and DE circRNAs were enriched in mitochondria and nucleus, which indicated that they may participate in the vital metabolism directly or indirectly, such as fatty acid metabolism. Subsequently, the protein–protein interaction (PPI) network was constructed and we got 8 key proteins, of which the matrix metalloproteinase-9 (MMP9; degree 5) draws our attention. Based on the 38 up-regulated circRNAs and 14 down-regulated circRNAs, we constructed competing endogenous RNA (ceRNA) networks, from which the has-miR-6794-5p has been identified to enriched in the up-regulated network and correlated with the circNFIX directly. At this point, we presented that the circNFIX and MMP9 may play a significant role by regulating fatty acid metabolism in breast cancer.

## Introduction

Breast cancer, the most threatening malignancy to female worldwide, caused about 2.3 million new cases according to the statistics in 2020 [1]. For the early-stage breast cancer, surgery remains preferred for local–regional therapy. As for systemic therapy, adjuvant endocrine therapy and adjuvant chemotherapy are advised for nearly all patients with invasive breast cancer [2]. Although these adjuvant treatments bring patients survival benefit, recurrence, metastases and treatment resistance are still unavoidable in some patients. Focusing on these problems, increased understanding of the molecular mechanisms in breast cancer is required urgently.

High-throughput next-generation sequencing (NGS) technologies, which allow sequencing millions of DNA or RNA sequence simultaneously, have a wide range of applications in diagnosis, treatments, and prognosis prediction for oncology [3–5]. Competing endogenous RNAs (ceRNAs) are RNAs that can compete for microRNAs binding and form regulatory networks across the transcriptome, thereby regulating varieties of pathological processes via distinct molecular mechanisms [6–8]. Recently, Wang et al. [9] reported that circRNA Hsa_circ_0005273 served as a sponging for miR-200-3p to facilitate breast cancer progression by regulating YAP1-hippo signaling pathway. Furthermore, Yu et al. [10] reviewed that circRNAs act as ceRNAs participate in lipid metabolism. The reported study not only indicated that the ceRNA cross-talk will affect the molecular functions and induce cancer progression but also represented a potential regulating mechanism in cancer. Therefore, RNA sequencing, which accurately reveals the gene expression profile, can be applied to investigate the molecular functions and mechanisms in breast cancer.

In our study, RNA sequencing was performed based on three tumor tissues (C) and their paired normal tissues (CP) from three breast cancer patients with different molecular subtypes. Subsequently, differentially expressed (DE) mRNAs, lncRNAs, and circRNAs were examined on the C and CP groups, followed by functional enrichment analysis, which were investigated through Gene Ontology (GO) categories and Kyoto Encyclopedia of Genes and Genomes (KEGG) analysis. The present study aims to explore the potential molecules for breast cancer diagnosis and treatment.

## Materials and methods

### Patients and tissue collection

The tissue samples were collected from three female breast cancer patients who underwent mastectomy at the First Affiliated Hospital of Jinan University, Guangzhou, between 2019 and 2020 and have no preoperative chemotherapy or radiation. All experimental procedures were approved by the Ethics Committee of the First Affiliated Hospital of Jinan University. The patients included in the present study signed inform consents. The three paired normal tissues used as control were obtained 5 cm away from the primary tumor sites. All patients were free of any other form of cancer and all their specimens were examined by pathologists before preservation in liquid nitrogen. Detailed patients’ information is shown in Supplementary Table 1.

### RNA extraction and quality control

Total RNAs were extracted from frozen tumor tissues and their paired normal tissues by TRIzol following the manufacturer's protocol. The preliminary RNA concentration and purity were evaluated with the NanoDrop 2000 Spectrophotometer (Thermo Scientific) and the precise quantification were performed by Qubit (ABI). Purified RNA was stored at −80°C.

### Library construction and sequencing

The RNA-seq library was generated using VAHTS Universal V8 RNA-seq Library Prep Kit for Illumina according to the manufacturer’s protocol. The quality of each library was controlled using an Agilent 2100 TapeStation (Agilent). The libraries were pooled and sequenced on an Illumina NovaSeq 6000 platform following to the manufacturer’s protocol and finally 150bp paired-end reads were generated.

### Quality control and alignment of RNA-sequencing reads

The raw RNA-sequencing reads were filtered to remove r-RNA using fastp to get the clean reads. Q20, Q30, and GC contents of the clean reads were calculated to evaluate the quality of sequencing. The clean reads were mapped to the reference genome using Hisat2. To defined the novel RNAs with or without coding potential, the transcript of each sample was assembled from mapped reads by StringTie. To estimate the expression levels of mRNAs and lncRNAs, the FPKM (Fragments Per Kilobases per Million reads) and TPM (Transcripts Per Million reads) were calculated through RSEM (http://deweylab.github.io/RSEM/) (Supplementary Table 2).

### Identification of DE transcripts

DE analysis was performed on C and CP using DESeq2. A *P*-value<0.05 and |log_2_ foldchange (FC)| >1 were considered as the thresholds for identifying significantly DE mRNAs, DE lncRNAs, and DE circRNAs. The volcano plots and heatmaps were drawn on DE mRNAs, DE lncRNAs, and DE circRNAs using the R package.

### Analysis of target mRNAs regulated by DE lncRNAs

DE lncRNAs-targeted mRNAs were predicted using the StarBase (http://starbase.sysu.edu.cn/) database. The lncRNAs–mRNAs interaction networks were constructed between DE lncRNAs and their targeted mRNAs by igraph package in R.

### Analysis of functional enrichment pathways

Analysis of the GO and KEGG enrichment pathways of DE mRNAs, DE lncRNAs, and DE circRNAs were performed by clusterProfiler package in R. The GO terms and KEGG pathways with the enriched gene count ≥ 2 and the significance threshold *P*≤0.05 were considered significant.

### Protein–protein interaction network construction

The STRING database (version 11.5) was used to predict the potential interactions among proteins translated by top 300 DE mRNAs. The threshold was selected as score ≥ 0.7. The protein–protein interaction (PPI) network was built by the STRING (https://string-db.org/).

### CircRNAs–miRNAs–mRNAs network construction

circRNA-–miRNA–mRNA interactions were predicted by miRanda (version 3.3a). The up-regulated and down-regulated circRNAs/mRNAs were selected to construct a ceRNA network by igraph package in R.

## Results

### Differential expression analysis

**Figure 1:**
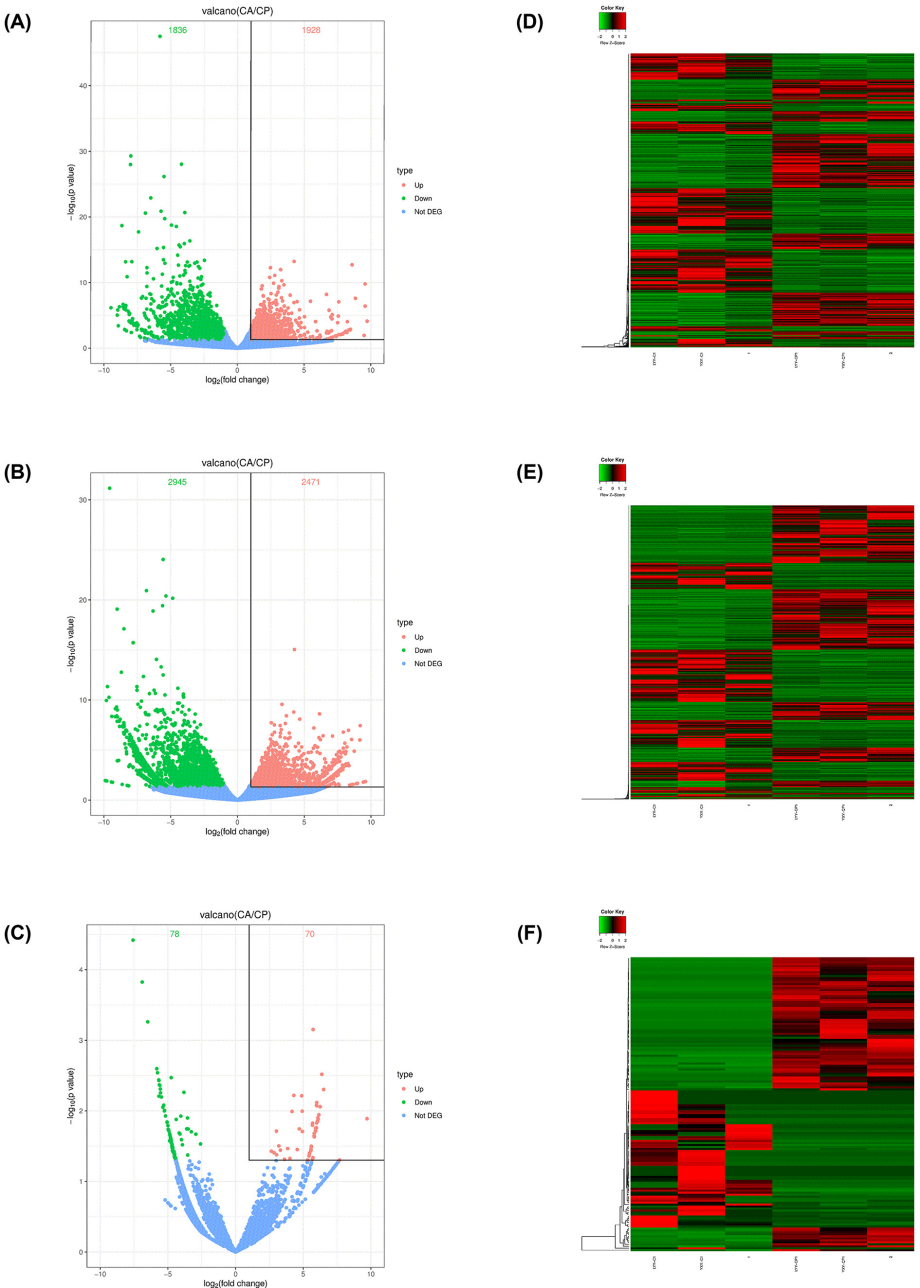
Analysis of DE mRNAs, lncRNAs, and circRNAs (**A–C**) Volcano plots of DE mRNAs (**A**), DE lncRNAs (**B**), and DE circRNAs (**C**). (**D–F**) Heatmaps of DE mRNAs (**D**), DE lncRNAs (**E**), and DE circRNAs (**F**). Red indicates up-regulation and green indicates down-regulation.

The volcano plots showed the DE genes were delimited clearly in the C and CP groups (Figure 1A–C). According to the screening criteria, a total of 3764 DE mRNAs were determined, of which 1928 were up-regulated and 1836 were down-regulated. DE lncRNAs analysis indicated 5416 deregulated lncRNAs, among those, significantly up-regulated and down-regulated RNAs were 2471 and 2945, respectively. For circRNAs, out of 148 dysregulated, 70 up-regulated and 78 down-regulated were observed. The hierarchical clustering analysis of DE mRNAs, DE lncRNAs, and DE circRNAs, which showed the expression profiles between the C and CP groups (Figure 1D–F), further explained the reliability of differential expression analysis.

### GO and KEGG enrichment analysis of DE mRNAs

**Figure 2:**
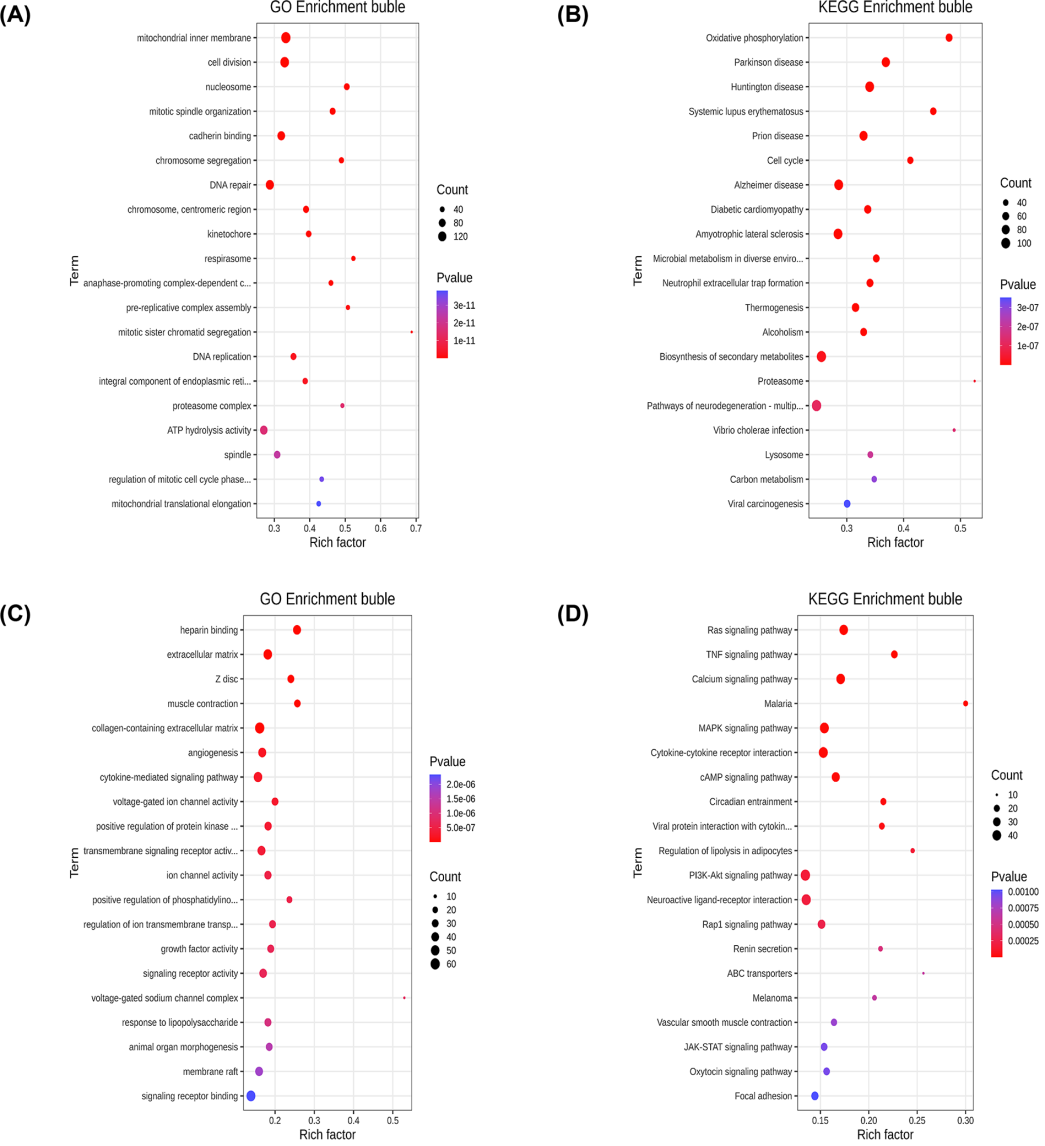
The GO and KEGG enrichment analysis of DE mRNAs (**A**) Top 20 GO terms enriched by up-regulated DE mRNAs. (**B**) Top 20 pathways enriched by up-regulated DE mRNAs. (**C**) Top 20 GO terms enriched by down-regulated DE mRNAs. (**D**) Top 20 pathways enriched by down-regulated DE mRNAs.

To predict the potential biological function of DE mRNAs, we performed GO and KEGG enrichment analysis to them. Figure 2 displayed top 20 enriched terms by up-regulated or down-regulated DE mRNAs. From the results, we found that most up-regulated DE mRNAs were related to cell division in biological process and the significantly enriched signaling pathway was biosynthesis of secondary metabolites (Figure 2A,B). For down-regulated DE mRNAs, several genes involved in cytokine-mediated signaling pathway (Figure 2C). Additionally, pathway analysis indicated that down-regulated DE mRNAs were mainly associated with MAPK signaling pathway and cytokine–cytokine receptor interactions (Figure 2D).

### PPI network

**Figure 3:**
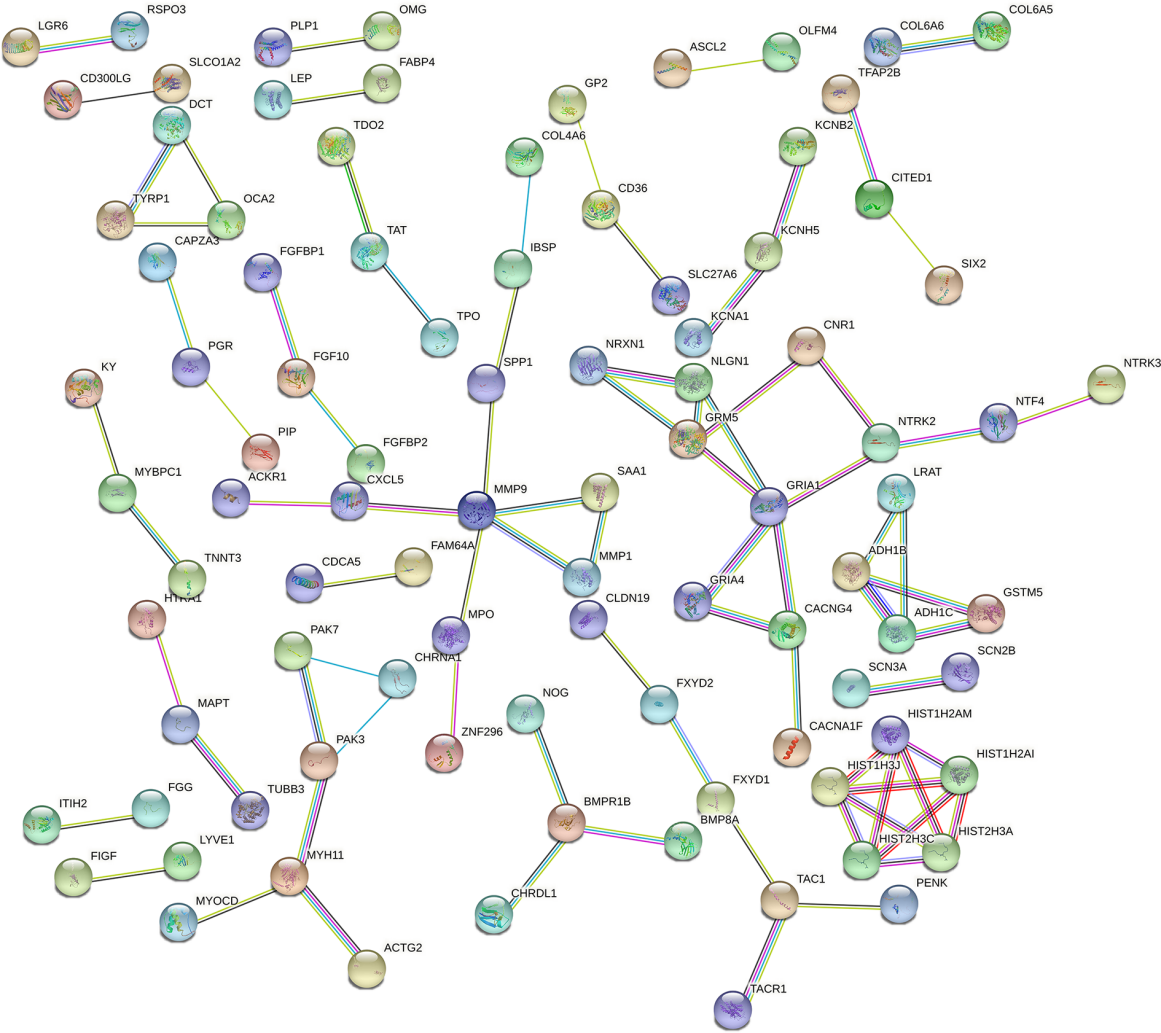
The PPI network in breast cancer using top 300 DE mRNAs The nodes in the network represent proteins and the color of edges indicates known or predicted interactions.

To better investigate the effect of protein interactions in breast cancer, a PPI network, which consisted of 93 nodes and 82 interaction pairs, was constructed using the STRING based on the top 300 DE mRNAs (Figure 3). The nodes with high degree can be regarded as key proteins, including GRIA1 (degree 5), matrix metalloproteinase-9 (MMP9; degree 5), GRM5 (degree 4), HIST1H2AI (degree 4), HIST1H2AM (degree 4), HIST1H3J (degree 4), HIST2H3A (degree 4), and HIST2H3C (degree 4). Since the proteins were involved in several essential signaling pathways, the dysregulation of them may cause the progression of breast cancer (Table 1).

**Table 1:** Top eight key proteins in PPI network

DE mRNAs	Express	Degree	Pathways
GRIA1	Down	5	cAMP signaling pathway
MMP9	Up	5	Lipid and atherosclerosis Transcriptional misregulation in cancer
GRM5	Up	4	Phospholipase D signaling pathway Calcium signaling pathway
HIST1H2AI	Up	4	Necroptosis Neutrophil extracellular trap formation
HIST1H2AM	Up	4	Necroptosis Neutrophil extracellular trap formation
HIST1H3J	Up	4	Transcriptional misregulation in cancer Neutrophil extracellular trap formation
HIST2H3A	Up	4	Transcriptional misregulation in cancer Neutrophil extracellular trap formation
HIST2H3C	Up	4	Transcriptional misregulation in cancer Neutrophil extracellular trap formation

### Functional enrichment analysis of lncRNAs and circRNAs

**Figure 4:**
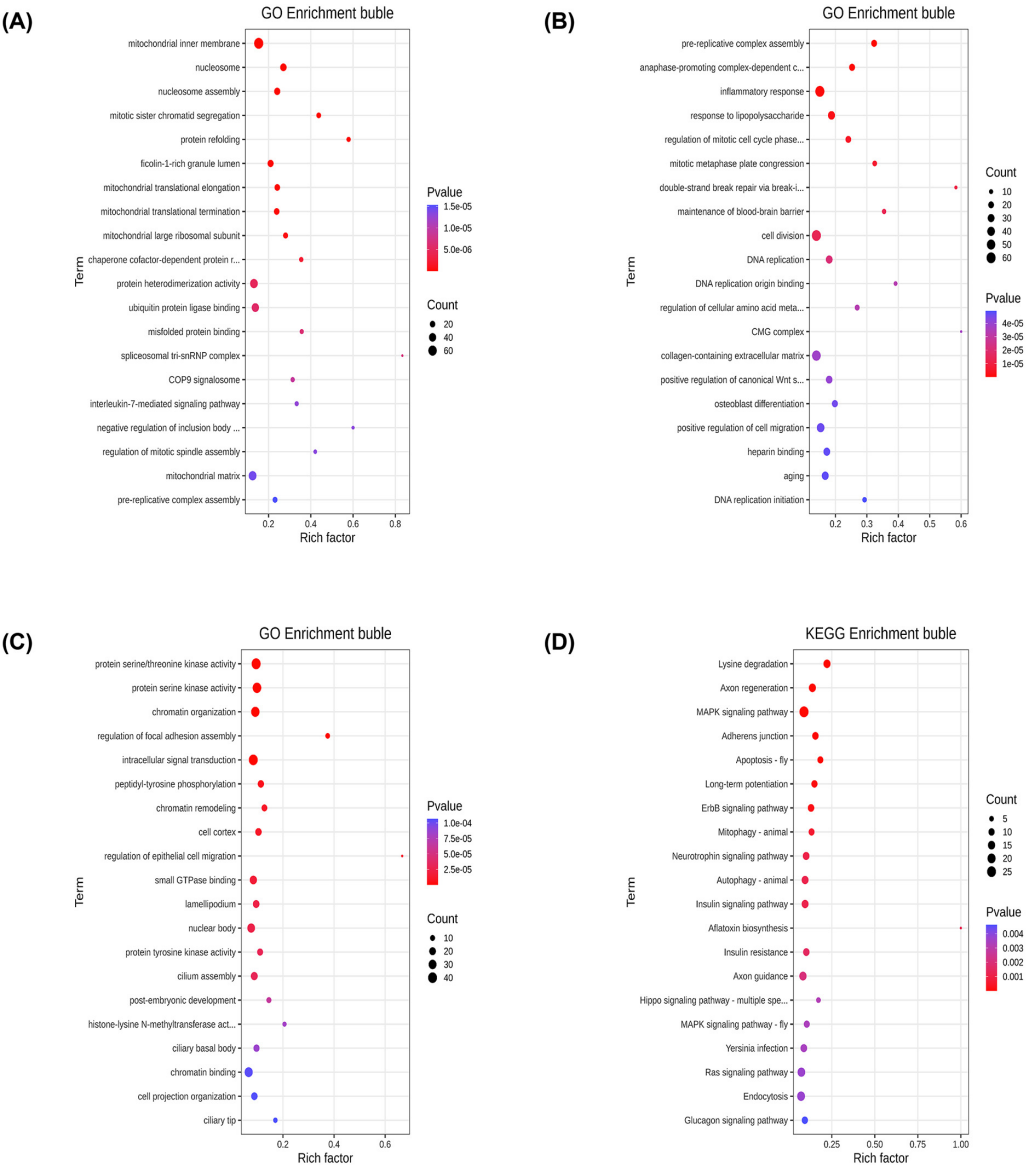
Bubble maps of enrichment analysis of lncRNAs and circRNAs (**A**) GO analysis of *cis*-target for DE lncRNAs. (**B**) GO analysis of *trans*-target for DE lncRNAs. (**C**) GO analysis of DE circRNAs. (**D**) Pathways analysis of DE circRNAs.

The diverse function of noncoding RNAs achieved by acting on the target genes in *cis* or *trans*. Depending on the location of the sites of transcription, *cis*-acting lncRNAs can activate or repress the expression of neighboring target genes in the linear genome [11,12]. The genes within a 100 kb of upstream or downstream in the DE lncRNAs were regarded as *cis*-acting lncRNAs, and the bubble diagram showed that the target genes were largely enriched in mitochondrial inner membrane (Figure 4A). Based on the absolute Pearson correlation coefficient over 0.99 and *P*-values less than 0.01, the possible *trans*-regulatory relationship was identified. The results suggested that the vast of majority *trans*-target mRNAs were involved in cell division (Figure 4B).

In order to explore the function of circRNA, GO and KEGG enrichment analysis were performed on DE circRNAs. As showed in Figure 4C, GO annotations revealed that many biological processes of DE circRNAs were related to intracellular signal transduction and chromatin organizations. KEGG pathway analysis showed that most relevant pathways of DE circRNAs were largely involved in MAPK signaling pathway (Figure 4D).

### LncRNAs–mRNAs interaction networks

**Figure 5:**
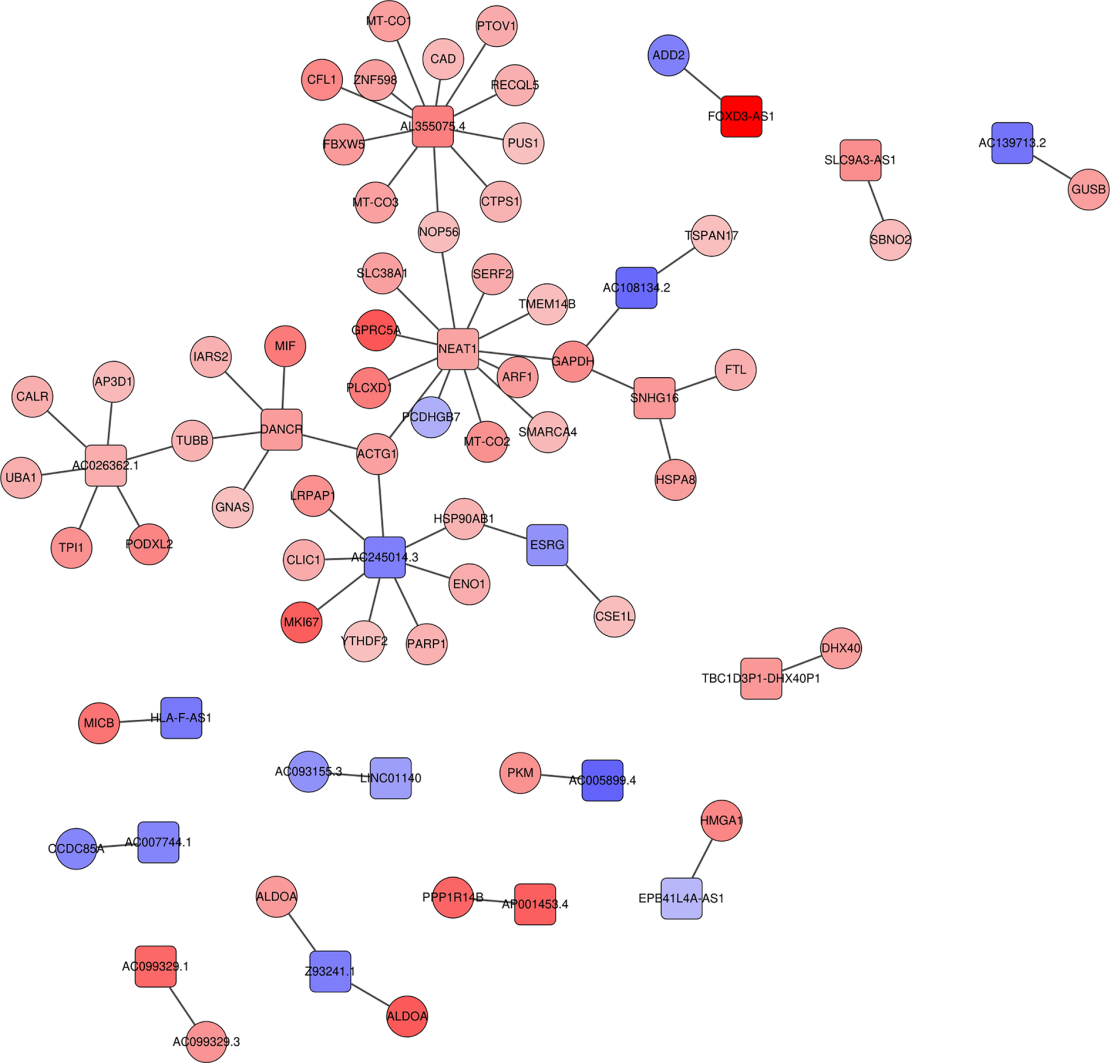
The lncRNAs–mRNAs interaction networks The interaction networks between DE lncRNAs and their targeted mRNAs. Quadrilateral represents the DE lncRNAs, circle represents the mRNAs.

In our study, 63 targeted mRNAs were predicted by DE lncRNAs using StarBase. To analyze the relationships between them, the lncRNAs–mRNAs interaction networks were constructed (Figure 5). Among the relationships, the number of up-lncRNAs were accounted for more than half and the proportion of up-mRNAs exceeded 90%, which suggested that the relationships in the interaction networks were mostly in the state of co-activation.

### ceRNA networks construction

**Figure 6:**
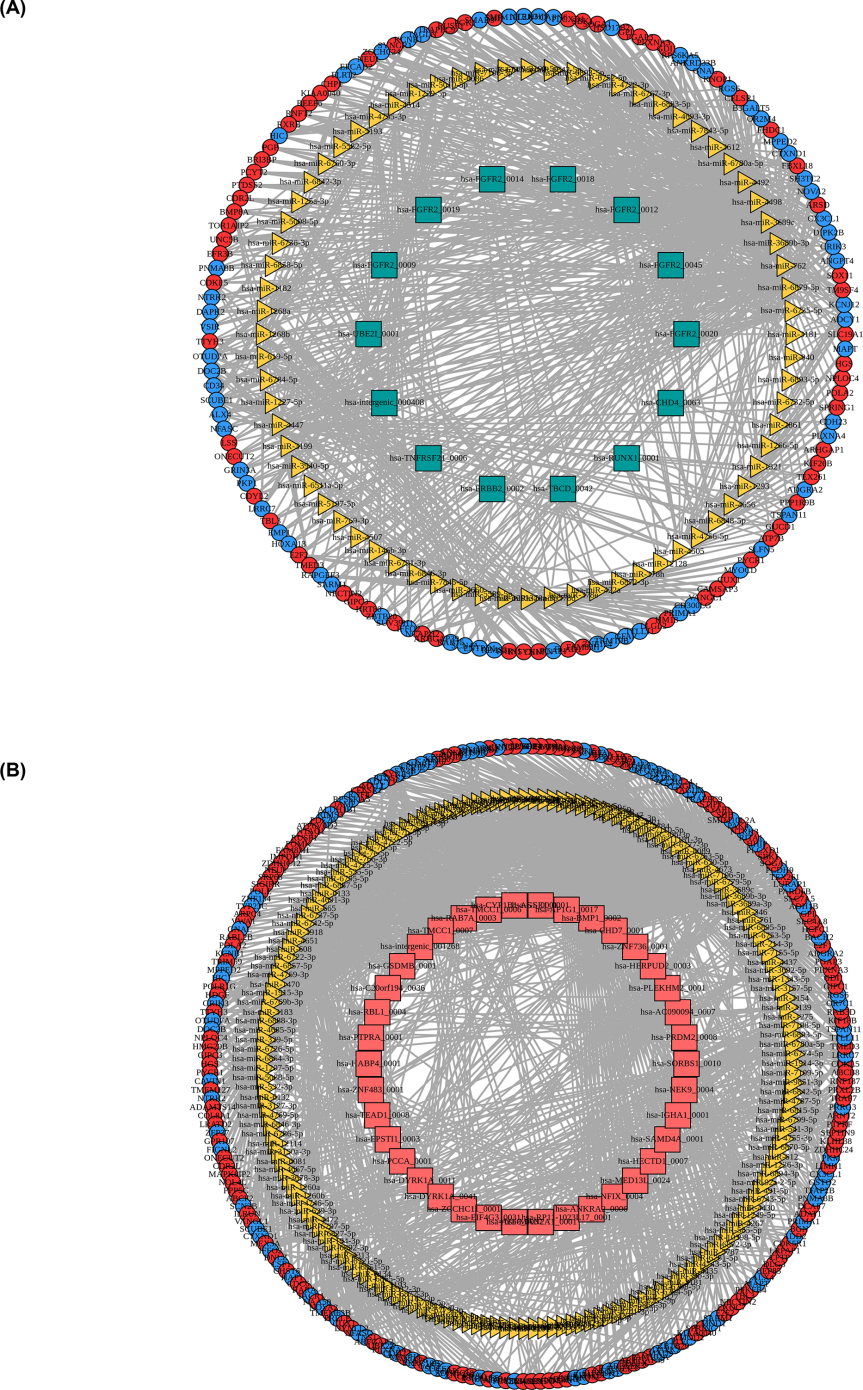
The circRNAs–miRNAs–mRNAs networks The ceRNA networks of down- (**A**) or up-regulated circRNAs (**B**). Green quadrilateral represents the down-regulated circRNA, yellow triangle represents the miRNA, red circle represents up-regulated mRNA, blue circle represents down-regulated mRNA, and red quadrilateral indicates the up-regulated circRNA.

circRNAs can act as endogenous RNAs of miRNAs to regulate the expression of mRNAs indirectly, further promoting or inhibiting the biological processes, such as proliferation, migration, or chemoresistance of breast cancer [13–16]. Based on the DE circRNAs and DE mRNAs, we predicted the miRNAs targets of DE circRNAs via miRanda and TargetScan, therefore constructing the circRNAs–miRNAs–mRNAs regulatory networks and found that has-miR-6794-5p was significantly enriched in the ceRNA network. A suppressed network is shown in Figure 6A, including 14 down-circRNAs, 72 miRNA, 150 down-mRNAs, and 125 up-mRNAs. Activated interaction pairs were obtained in Figure 6B, of which 38 up-circRNAs, 175 miRNA, 197 down-mRNAs, and 242 up-mRNAs were included.

## Discussion

RNA sequencing, which can be applied to identify the tumor driver genes, not only brought incredible changes and advantages in the field of tumor [17–19], but expands understanding and provides potential treatment strategies for some special types of tumors, such as inflammatory breast cancer and juvenile papillomatosis with co-existing breast carcinoma [20,21]. In our study, we found 3764 DE mRNAs, 5416 DE lncRNAs, and 148 DE circRNAs by RNA sequencing in three breast cancer tissues compared with their paired normal tissues. Above all, enrichment analysis indicated that DE lncRNAs and DE circRNAs were mainly enriched in mitochondria and nucleus. Apparently, nucleus is the control center of various cell metabolisms and mitochondria produce ATP for them. Recently, fatty acid oxidation (FAO) has been proved to be a crucial energy pathway in metastatic breast cancer, and the key enzyme of FAO exist in mitochondria, such as CPT1 (carnitine palmitoyl transferase 1), limit the rate of reaction thus restricting the proliferation of malignant cells [22–24]. Moreover, ATP citrate lyase (ACLY), a key enzyme responsible for *de novo* fatty acid synthesis, was considered to enhance triple-negative breast cancer (TNBC) stemness and metastasis via miR-206/ITGA2/ACLY-CCND1 signaling axis [25]. It follows that the exploration of noncoding RNAs, which can regulate the key enzyme of fatty acid metabolism, may reveal the mechanisms of the breast cancer progression in the future.

In our study, we found that up-circRNA NFIX, which directly correlated with has-miR-6794-5p, was a significant role in the circRNAs–miRNAs–mRNAs network. Previous evidence demonstrated that circNFIX can promote progression in several types of cancer, such as glioma, lung cancer, and pituitary adenoma [26–28]. Recent study has reported that circNFIX can function as a ceRNA of has-miR-3064-5p to facilitate the cell growth, migration, and invasion of hepatocellular carcinoma (HCC), further enhance glutaminolysis [29]. To our knowledge, the role of circNFIX in breast cancer is still unknown. Since fatty acid metabolism can induce breast cancer progression as well as therapy resistance [30–34], whether circNFIX regulates fatty acid metabolism in breast cancer is worthy further investigation in the future.

Additionally, our PPI network identified several hub proteins from vital DE mRNAs, which were speculated to be potential factors in regulating breast cancer. For example, MMP9, an important enzyme to the degradation of extracellular matrix (ECM), was considered to be an onset of invasion or metastasis in breast cancer [35–37]. Furthermore, MMP9 was suggested can be regulated by free fatty acids receptor 1 (FFA1), which indicated that fatty acid metabolism may be a potential mechanism of MMP9 activity [38]. In addition, Coilly et al. [39] identified MMP9 as a predictive factor for poor prognosis of nonalcoholic fatty liver. Kwapisz et al. [40] reported that fatty acid can activate MMP9 and further induce epithelial–mesenchymal transition (EMT), which can even lead to the occurrence of HCC. Thus, we predicted that MMP9 is related to fatty acid in our study. Based on the previous study, the mechanism research, which about MMP9 promotes breast cancer progression by regulating fatty acid metabolism, possibly serving as a therapeutic strategy in breast cancer.

Elevated expression of MMP9 was correlates with more aggressive subtypes of breast cancer, such as TNBC and HER2-positive breast cancer [41]. In our study, two pairs of HER2-positive breast cancer and one pair of TNBC samples were used for RNA-sequencing and further analyses revealed that MMP9 was one of the most up-regulated expressed mRNA and may promote breast cancer progression by regulating fatty acid metabolism. As the most aggressive molecular subtypes in breast cancer, TNBC and HER2-positive breast cancer remained obstacles in the therapy of recurrence, metastasis, or chemotherapy resistance. The metabolic regulatory roles of MMP9 and the upstream regulatory mechanism of the elevated expression of MMP9 in TNBC and HER2-positive breast cancer require further study. In the present study, we found that circNFIX may act as a ceRNA through regulating MMP9 expression and fatty acid metabolism in breast cancer, which may be a novel mechanism of elevated MMP9 expression.

In conclusion, our study provides insights into the molecular mechanisms of breast cancer by analyzing the expression profiles of mRNAs, lncRNAs, and circRNAs. circNFIX and MMP9 may play a unique role by regulating fatty acid metabolism in breast cancer, which suggest a potential therapy in the future. However, our study has lack of analysis of miRNAs to provide a whole-transcriptome sequencing for breast cancer. In addition, based on our present study, further experimental validations are required to perform.

## Supplementary Material

Supplementary Tables S1-S2

## Data Availability

The data generated or analyzed to support the current study are included within the article.

## Abbreviations

ACLY: ATP citrate lyase
ceRNA: competing endogenous RNA
DE: differentially expressed
FAO: fatty acid oxidation
FC: foldchange
FPKM: fragments per kilobases per million
GC: The proportion of the bases G and C account for the total bases A, T, C, G.
GO: gene ontology
HCC: hepatocellular carcinoma
KEGG: Kyoto Encyclopedia of Genes and Genomes
MMP9: matrix metalloproteinase-9
NGS: next-generation sequencing
PPI: protein–protein interaction
TNBC: triple-negative breast cancer
TPM: transcripts per million reads
