# Supplementary material for: Differential expression profile of mRNAs, lncRNAs, and circRNAs reveals potential molecular mechanism in breast cancer
Source: Biosci Rep. 2022 Jul 29;42(7):BSR20220645. doi: 10.1042/BSR20220645 (PMC9338430; doi:10.1042/BSR20220645)
Supplement: Supplementary Tables S1-S2 [file BSR-2022-0645_supp.pdf]

Supplementary Table 1. Patients' information

	Year	HR	HER-2	Ki-67(%)	TNM	Grade
**Patient 1**	39	-	-	65	T3N0M0	III
**Patient 2**	70	-	+++	30	T1N0M0	II
**Patient 3**	49	+	+++	70	T2N3M0	II

Supplementary Table 2. Summary of RNA sequencing data obtained in the study

Sample	Raw Reads	Raw Bases	rRNA	Clean Reads	Clean Bases	Clean Ratio	Q20	Q30	GC
C1	99613588	14761035187	771668 (0.77%)	97934906	14507655458	99%	98%	95%	42%
CP1	111185506	16438867314	1153060 (1.04%)	108563450	16046939359	99%	98%	95%	40%
C2	120718204	17869084800	1452853 (1.20%)	116646934	17259986125	98%	97%	93%	43%
CP2	118099104	17496802551	685611 (0.58%)	114940884	17021730149	98%	97%	93%	40%
C3	114398138	16596876883	2660047 (2.33%)	109943816	15944408010	99%	98%	94%	44%
CP3	109640150	15730199790	815713 (0.74%)	107220616	15371021444	99%	98%	94%	40%
